# The Role of the Leukemia Inhibitory Factor (LIF) — Pathway in Derivation and Maintenance of Murine Pluripotent Stem Cells

**DOI:** 10.3390/genes2010280

**Published:** 2011-03-09

**Authors:** Urs Graf, Elisa A. Casanova, Paolo Cinelli

**Affiliations:** 1 Institute of Laboratory Animal Science, University of Zurich, Winterthurerstrasse 190, CH-8057 Zurich, Switzerland; E-Mails: urs.graf@access.uzh.ch (U.G.); ecasanova@access.uzh.ch (E.A.C.); 2 Molecular Life Sciences Graduate School, University of Zurich, Winterthurerstrasse 190, CH-8057 Zurich, Switzerland; 3 Center for Applied Biotechnology and Molecular Medicine, University of Zurich, Winterthurerstrasse 190, CH-8057 Zurich, Switzerland

**Keywords:** LIF, STAT3, pluripotency, embryonic stem cells, induced pluripotent stem cells

## Abstract

Developmental biology, regenerative medicine and cancer biology are more and more interested in understanding the molecular mechanisms controlling pluripotency and self-renewal in stem cells. Pluripotency is maintained by a synergistic interplay between extrinsic stimuli and intrinsic circuitries, which allow sustainment of the undifferentiated and self-renewing state. Nevertheless, even though a lot of efforts have been made in the past years, the precise mechanisms regulating these processes remain unclear. One of the key extrinsic factors is leukemia inhibitory factor (LIF) that is largely used for the cultivation and derivation of mouse embryonic and induced pluripotent stem cells. LIF acts through the LIFR/gp130 receptor and activates STAT3, an important regulator of mouse embryonic stem cell self-renewal. STAT3 is known to inhibit differentiation into both mesoderm and endoderm lineages by preventing the activation of lineage-specific differentiation programs. However, LIF activates also parallel circuitries like the PI3K-pathway and the MEK/ERK-pathway, but its mechanisms of action remain to be better elucidated. This review article aims at summarizing the actual knowledge on the importance of LIF in the maintenance of pluripotency and self-renewal in embryonic and induced pluripotent stem cells.

## 1. Introduction

**Figure 1:**
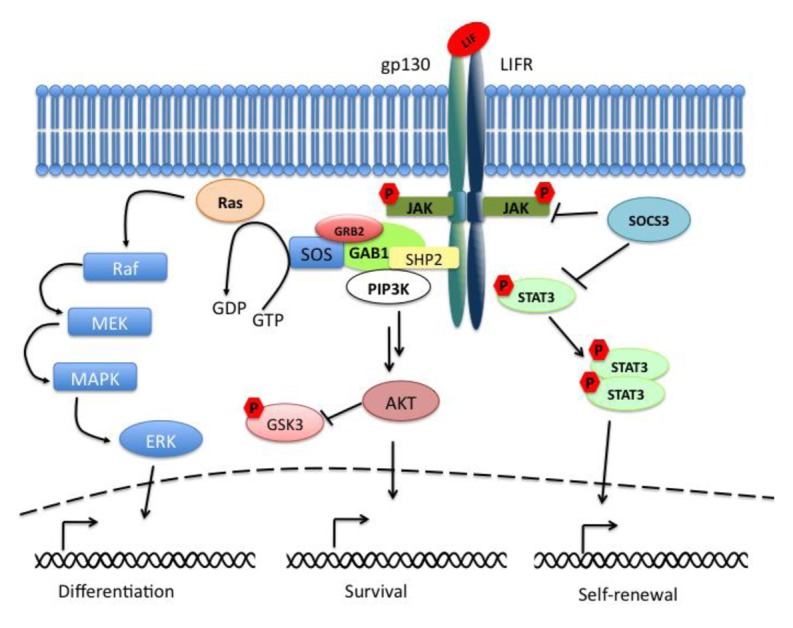
Schematic representation of the LIF-pathway.

The Janus kinase signal transducer and activator of transcription (JAK-STAT) pathway mediates the transmission of information received from extracellular polypeptide signals, through transmembrane receptors and directly targets gene promoters without needing second messengers. Leukemia inhibitory factor (LIF) belongs to the family of interleukin (IL)-6-type cytokines, which signal through the common receptor subunit gp130 in association with a ligand-specific receptor subunit, the low-affinity LIF receptor (LIFR). The binding of LIF to the LIFR induces its heterodimerization with gp130. The formation of this complex results in the activation of the receptor-associated Janus kinases (JAKs), in the phosphorylation of receptor docking sites, and finally in the recruitment of Src homology-2 (SH2) domain containing proteins such as STAT3 (signal transducer and activator of transcription 3). When bound to the receptor, STAT3 molecules are phosphorylated on tyrosine 705 (Tyr705) residues and dimerize with another phosphorylated STAT3. The dimers are then translocated to the nucleus in a regulated manner where they bind to promoters and enhancer regions of their target genes (Figure 1). The suppressor of cytokine signaling 3 (SOCS3) is an important negative regulator of the LIF/STAT3-pathway whose expression is directly driven by STAT3. SOCS3 plays a critical role in many cell types and tissues in which the LIF-pathway is active.

In parallel to the activation of the STAT3-pathway, the binding of LIF to the LIFRβ/gp130 receptor leads to the activation of the mitogen-activated protein kinase (MAPK) and the phosphatidylinositol-3 phosphate kinase (PI3K) pathways. Together with the JAK/STAT3-pathway they are essential components for the regulation of biological responses in embryonic stem cells (ESCs). Active gp130 receptor can associate with the protein tyrosine phosphatase SHP-2 [1], a positive effector of the MAPK signaling cascade, and lead to recruitment of Gab1. The complex gp130/SHP-2/Gab1 initiates a phosphorylation cascade that culminates in the activation of the ERK1 and ERK2 kinases [2] (Figure 1).

Last but not least, LIF binding to the LIFRβ/gp130 receptor induces the activation of members of the class IA family of phosphatidylinositol-3 phosphate kinases (PI3Ks). PI3Ks mediate signal transduction through downstream effector molecules including the serine/threonine protein kinase B (PKB, also known as AKT) that has been implicated in many cellular processes like regulation of the cell cycle progression, cell death, adhesion, migration, metabolism and tumorigenesis (for review see [3]). GSK3β (glycogen synthase kinase 3β) is a common target of the PI3K/AKT-pathway and of the Wnt-pathway. These pathways control GSK3β phosphorylation thereby regulating its inactivation (Figure 1).

## 2. The LIF Signaling Pathway and Pluripotency

### 2.1. The Role of LIF/STAT-Pathway during Preimplantation Development

Preimplantation embryos express LIF, LIFβR and gp130 mRNA highlighting a possible function of the LIF/STAT3-pathway *in vivo*. However, studies on knockout mouse models indicate that at least the early embryonic development can occur also in the absence of some components of the LIF-pathway but many of these mouse models then display later on during development or during adulthood many different disorders (Table 1). *Lif*−/− embryos, for instance, develop normally into adulthood [4,5] but even though *Lif*−/− females are fertile, their blastocysts cannot implant due to the absence of LIF production by the uterus. This is of note because *p53*−/− mice display the same implantation defects as *Lif*−/−. Interestingly, development of both *p53*−/− and *Lif*−/− embryos can be rescued by transferring them to a wild-type female [6]. Another example are embryos carrying mutations on the LIFβR, which also develop to term but have alterations in the fetal liver metabolism, in the formation of bones, and in the survival of astrocytes and motor neurons in the spinal cord and brain stem [7,8]. Last but not least, *gp130*−/− embryos are able to develop to blastocysts and to implant but die between day 12 to 16 of embryogenesis with cardiac, hematopoietic and neuronal disorders [9].

These findings demonstrate that the LIF-pathway is dispensable *in vivo* during preimplantation. However, ESCs are believed to be the *in vitro* counterpart of the transient pool of pluripotent cells present in the early epiblast, but are in contrast to the cells of the embryos, LIF-dependent.

In mice, a possible explanation for this discrepancy is the existence of a phenomenon called diapause. Mice can temporarily arrest embryogenesis at the blastocyst stage thereby overcoming suboptimal conditions for reproduction. During diapause the embryos develop to the hatched blastocyst stage but stop their development, remaining unimplanted in the uterus. During this period that can persist for weeks, the epiblast cells have to be maintained as pluripotent until the development of the embryo is restored. Interestingly, diapause-arrested embryos carrying mutations on the LIFβR and the gp130 receptors fail to restore normal embryogenesis [10]. This highlights the absolute requirement for LIF/gp130 signaling in the epiblast during diapause. ESCs were first established from diapause embryos [11] and this could represent a potential explanation why ESCs are LIF dependent.

**Table 1.** Effect of gene targeting and overexpression of leukemia inhibitory factor (LIF)— pathway members *in vivo* and in ESCs.

**Gene/Protein**	**KO/overexpression in vivo**	**KO/overexpression in ESCs**	**Literature**
LIF	Viable, *Lif*−/− females are fertile, but their blastocysts cannot implant. LIF overexpression is lethal and leads to the absence of differentiated mesoderm. Embryos do not undergo normal gastrulation.	*Lif*−/− ESCs cannot be derived under classical culture conditions.	[12] [13] [14] [4]
gp130	*gp130*−/− embryos can survive diapause but the epiblast compartment is not maintained. They die between E12.5 and E16 because of developmental defects.	*gp130*−/− ESCs cannot be derived under classical culture conditions. Modest gp130 overexpression enhances self-renewal in the absence of exogenous LIF, greater levels of receptor expression increases differentiation.	[9] [10] [15] [13]
LIFRβ	*Lifr*−/− embryos die around the time of birth with severe deficits in motor neuron and glial cell populations. *Lifr*−/− embryos do not survive prolonged diapause.	*Lifr*−/− ESCs cannot be derived under classical culture conditions.	[16] [8] [10] [13]
STAT3	*Stat3* null embryos undergo normal initial formation and growth of the epiblast but gastrulation is disrupted.	*Stat3* null ESCs can only be derived under 2i conditions. STAT3 overexpression is sufficient to maintain ESCs pluripotent in absence of LIF.	[17] [18] [19]
SOCS3	Essential for maintenance of murine ESC pluripotency and trophoblast differentiation.	*Socs3* null cells show enhanced activation of STAT3 and SHP2.	[20] [21] [22] [23] [24] [25]
JAK1	JAK1 is essential for the differentiation of the trophoblast. *Jak*−/− mice die within the first 24 hours after birth.	JAK1 is essential for LIF signaling in ESCs.	[25] [26] [27] [28]
JAK2	*Jak2*−/− embryos are anemic and die around day 12.5 postcoitum	*Jak2*−/−ESCs are competent for LIF signaling. JAK2 plays a role in early lineage decision.	[29] [26]
Shp2	*Shp2* null blastocysts initially develop normally, but they subsequently exhibit inner cell mass death, reduced number of trophoblast giant cells, and failure to yield trophoblast stem cell lines. Homozygous null mutants die at embryonic day 10.5	Deletion of *Shp2* in mouse ESCs inhibits differentiation. *Shp2* null ESCs show increased STAT3 phosphorylation after LIF stimulation.	[30] [31] [32]

### 2.2. Establishing and Maintaining Pluripotent Stem Cells in vitro

Pluripotent stem cells harbor two important properties: the capacity of indefinite self-renewal *in vitro* and the ability to contribute to the formation of all cells of an adult organism including the generation of functional gametes for genome transmission. Due to their pluripotent state these cells can be used for various applications, like the generation of knockout or transgenic animals, and potentially as a cell source for cell therapy in regenerative medicine.

Two types of murine pluripotent stem cells can be derived. The embryonic stem cells (ESCs) which are isolated from the inner cell mass (ICM) of blastocyst stage embryos [11,33] and the induced pluripotent stem cells (iPSCs), which are generated by reprogramming somatic cells using gene transfection [34]. Murine pluripotent ESCs are characterized by the expression of specific cell surface glycoproteins such as the stage-specific embryonic antigen 1 (SSEA-1) [35] as well as by the presence of transcription factors like OCT3/4 [36,37] and Nanog [38,39]. Furthermore, ESCs express elevated levels of alkaline phosphatase and have high telomerase activity [40].

It is a combinatorial activity of different signaling pathways that orchestrates the maintenance of ESCs in a pluripotent state. For example, it has been shown that bone morphogenetic protein (BMP) enhances self-renewal and pluripotency of ESCs in the presence of LIF [41]. Moreover, activation of the canonical WNT-pathway was shown to maintain the undifferentiated phenotype in both mouse and human ESCs, and to sustain expression of the pluripotency markers like OCT4, REX1 (zinc-finger protein-42; ZFP42) and Nanog in the absence of LIF [42]. Furthermore, nutrients and other environmental cues, like amino acids [43] and inositols [44], are also involved in the regulation of early mouse embryos and ESCs survival.

#### 2.2.1. The Role of LIF in Pluripotent Stem Cells

Murine ESCs are routinely isolated and maintained *in vitro* by using mitotically inactivated embryonic fibroblasts (feeders) and fetal calf serum. In 1988, LIF was identified as a paracrine signal produced from the feeders preventing stem cell differentiation while promoting ESC self-renewal [45–47]. In the following years, ESC culture conditions have been constantly improved, so that for example, feeders could be substituted through addition of LIF to the medium whereas serum could be replaced through addition of BMP [48].

Only recently, new defined culture conditions free from feeders, serum and cytokines, were established by using a combination of small-chemical molecules, which inhibit the mitogen-activated protein kinase (MAPK)/extracellular signal-related kinase (ERK1/2) and the glycogen synthase kinase 3 (GSK3). Under these conditions (2i), it was possible to maintain self-renewal in the absence of LIF/STAT3 stimulation as shown by the fact that in ESCs cultivated under these conditions neither STAT3 nor SOCS3 activation was detected. Moreover, the presence of 2i allowed the establishment of *Stat3* null cells [19]. Nevertheless, addition of LIF to the 2i culture conditions maximizes clonogenic self-renewal of ESCs, highlighting once more the importance of LIF in ESC maintenance.

As mentioned before, in mouse ESCs LIF does not only activate the STAT3-mediated cascade, but also induces the PI3K-pathway and the ERK-pathway. The PI3K-pathway is involved in the maintenance of pluripotency [49–51], whereas the ERK-pathway along with the FGF-pathway induces pro-differentiative programs [52,53]. These observations strongly suggest that several LIF induced pathways are concomitantly necessary for the maintenance of ESC identity.

The importance of the LIF-pathway components in derivation and maintenance of ESCs has extensively been studied. The LIFR receptor is not sufficient to mediate the signal to maintain ESC self-renewal, whereas gp130 is [54], indicating that gp130 is the main component of the activated LIFR/gp130 receptor. Moreover, ESCs can be derived and maintained using a combination of interleukin-6 and soluble interleukin-6 receptor (IL-6/sIL-6R) [55,56], which induces the homodimerization of gp130 and the activation of signaling processes that sustain self-renewal of ESCs. These studies indicate that signals mediated from gp130 are sufficient for self-renewal. Moreover, STAT3 activation is essential for LIFR/gp130-mediated ESC self-renewal [54] and its constitutive expression is sufficient for keeping ESCs pluripotent in the absence of LIF [18]. Nevertheless, the downstream components activated by LIF are still not completely understood and especially how LIF is mediating the activation of the MAPK- and PI3K-pathways has not yet been clarified.

#### 2.2.2. The LIF/STAT-Pathway in Embryonic Stem Cells

STAT3 activation has previously been shown to be necessary and sufficient to maintain self-renewal of mouse ESCs in the presence of serum or serum replacement [18,54,57,58]. Matsuda and colleagues used a chimeric STAT3-modified estrogen receptor (STAT3MER) composed of the entire coding region of STAT3 and the ligand-binding domain of the estrogen receptor. Dimerization of the chimeric STAT3 was activated after treatment with the estrogen derived 4-hydroxy-tamoxifen (OHT). ESCs cultivated in the presence of OHT were able to self-renew in the absence of LIF. The importance of STAT3 in ESCs is further supported by the observation that inactivation of Stat3 by a dominant interfering mutant (STAT3F) in LIF-maintained ESCs promotes spontaneous differentiation [54]. In a recent study, by using the same STAT3 inducible system, we were able to generate ESCs from the non-permissive FVB/N mouse strain in the presence of 4-OHT with high efficiency [58], highlighting the importance of this factor also in the derivation of ESCs from the ICM of the blastocysts.

Even though these observations provide evidence for the role of STAT3 as an important component of the LIF-dependent self-renewal in ESCs, the downstream target genes of activated STAT3 have remained elusive. Several studies based on chromatin immunoprecipitation (ChIP) analysis or on microarray technology have recently been performed aiming at the identification of these genes [58–63]. The transcription factor c-MYC was described to play an important role in self-renewal by functioning as a key target of LIF/STAT3 signaling [62]. Constitutive expression of *c-Myc* renders self-renewal independent of LIF [62], a phenomenon that was described also for other genes involved in the maintenance of pluripotency, such as *Nanog* [39], *Klf2* [64], *Pem/Rhox5* [58,65] and *Pramel7* [58,66]. On the other hand expression of a dominant negative form of c-MYC promotes differentiation [62]. c-MYC is a transcription factor that controls many different biological processes, such as cell proliferation, growth, differentiation and apoptosis. Moreover, c-Myc is, together with OCT3/4, KLF4, and SOX2, one of the transcription factors able to reprogram somatic cells into undifferentiated iPSCs [34].

A link between STAT3 and other pluripotency related genes has also been described. A recent study showed that 55% of the putative STAT3 target genes display binding sites for Nanog, and 41% of the putative Nanog target genes display binding sites for STAT3 [67]. These results highlight the fact that these transcription factors co-regulate the expression of a large number of target genes involved in the maintenance of the undifferentiated state in ESCs. Another study identified 24 STAT3 target genes which showed binding sites for STAT3 and/or Nanog. Knockdown of these genes increased differentiation of ESCs in LIF-supportive conditions, confirming their contribution to pluripotency [63]. Some of the identified genes were already known to be STAT3 targets, like *Pim-1* and *Pim-3* kinases [68] and two genes of the Krüppel-factors family, namely *Klf4* and *Klf5*. *Klf4* was recently proposed as a downstream target of LIF/STAT3 also by other research groups [64,69]. LIF/STAT3 selectively induces *Klf4* but its overexpression is not sufficient to sustain prolonged ESC self-renewal in the absence of LIF or STAT3 [64].

We could recently identify *Pramel7* as a new STAT3 target gene [66]. Ablation of Pramel7 in ESCs induces differentiation, while its overexpression is sufficient to support long-term self-renewal in the absence of exogenous LIF and suppresses differentiation. LIF-mediated *Pramel7* expression involves both STAT3-dependent transcriptional regulation and PI3K-dependent phosphorylation of GSK3. Pramel7 expression in turn confers constitutive self-renewal and prevents differentiation through inactivation of ERK phosphorylation. Moreover, Pramel7 knockdown induced ESC differentiation in the presence of LIF and upon forced STAT3 activation, indicating that Pramel7 is a mediator of the LIF- and STAT3-dependent maintenance of pluripotency in ESCs. Therefore, Pramel7 represents a central and essential factor in the signaling network regulating pluripotency and self-renewal in ESCs [66].

A further component of the LIF/STAT3-pathway is SOCS3, a STAT3-dependent repressor that attenuates LIF signaling. Different studies showed that SOCS3 is essential for maintenance of murine ESC pluripotency and for trophoblast differentiation [21–25]. LIF binding to the LIFR/gp130 heterodimer induces auto-phosphorylation of associated JAK1 (pJAK1), which subsequently phosphorylates the receptor tyrosines. Upon binding to phosphorylated tyrosines on gp130, STAT3 becomes phosphorylated, dimerizes and translocates to the nucleus where it induces transcription of SOCS3. SOCS3 binds to phosphorylated tyrosine 757 on gp130 and may undergo a conformational change. SOCS3 then binds to pJAK1 and the complex is released from the receptor complex. This inhibits further STAT3 activation, as tyrosines within the intracellular domain are no longer being phosphorylated by JAK1. SOCS3 bound pJAK1 promotes the proteasomal degradation of pJAK1 [20].

#### 2.2.3. The LIF/ERK/MEK-Pathway

ERK has been shown to regulate early differentiation *in vivo* and *in vitro* [52,70]. Upon LIF stimulation, both ERK1 and ERK2 are activated through phosphorylation by SHP-2 [71]. Nevertheless, suppression of SHP-2/ERK does not affect the propagation of stem cells, but on the contrary enhances ESCs self-renewal [71]. This indicates that SHP-2/ERK signaling activation is necessary for the normal differentiation processes. ERK signaling is also involved in the regulation of proliferation and survival of somatic cells [72] and in the regulation of early preimplantation development. Phosphorylated ERK has been detected from the 2-cell stage embryo until the blastocyst stage [73]. Incubation of 2-cell stage embryos with an ERK inhibitor results in a developmental arrest at the four-cell stage embryo, however the development can be restored once the inhibitor is removed [74]. This confirms that the ERK-pathway is required for progression of early cell division cycles in the preimplantation embryo. Entrance into the G1 phase of the cell cycle is a prerogative for cell differentiation. During the development from the 2-cell to the 8-cell stage embryo, ERK signaling seems to be essential in the G2/M transition [74]. This is in contrast to what is happening in many mammalian cells, where ERK activity is essential for the cell cycle progression from G0/G1 to S phase [75].

Interestingly the ERK-pathway may be able to inhibit the JAK/STAT3-pathway at the level of STAT3. It has been shown that ESCs knocked out for *Shp2* phosphatase after LIF stimulation showed an increased phosphorylation of STAT3 when compared to wild-type cells [31]. This data supports the evidence that the two pathways seem to converge and thereby determine the choice between self-renewal and differentiation (Figure 1).

#### 2.2.4. The LIF/PI3K-Pathway

In ESCs the LIF/PI3K-pathway was initially implicated in the control of proliferation [76,77]. Later it could be demonstrated that LIF-induced PI3K signal activation is also involved in ESC self-renewal [51]. Incubation of ESCs with LIF and LY294002, a small chemical inhibitor of PI3K, showed a reduced ability of LIF to promote self-renewal and a reduction of alkaline phosphatase positive cells. Since under these conditions no changes in the phosphorylation levels of STAT3 could be detected, the observed loss of self-renewal and the consequent differentiation of the cells were explained by an increase in ERK phosphorylation upon LIF stimulation [51]. These findings are consistent with other studies where it was reported that PI3K plays a role in negatively regulating ERK activity in ESCs [78]. Paling *et al.* demonstrated that self-renewal was restored after incubation with both ERK and PI3K inhibitor, therefore confirming that the regulation of ERK activity by PI3K signaling contributes to the determination of ESCs self-renewal [51].

The importance of PI3K/AKT signaling in the regulation of ESCs is also corroborated by the fact that expression of a myristoylated active form of AKT liberates ESCs from LIF-dependence. These cells showed no alteration in the level of phosphorylated STAT3 compared to the control cells, but interestingly exhibited increased ERK phosphorylation [50]. These findings are in contrast with the results from Paling *et al.* [51] indicating that increased ERK phosphorylation upon PI3K inhibition leads to differentiation.

Wnt blocks proteasome-mediated degradation of β-catenin through the inhibition of GSK3β (for review see [79,80]). Additionally, β-catenin signaling has also been postulated to be involved in the AKT-mediated maintenance of the undifferentiated state. Although in transgenic ESCs expressing a constitutive active form of AKT, GSK3β phosphorylation was enhanced, β-catenin signaling was not activated. It was therefore concluded that AKT-dependent maintenance of the undifferentiated state in ESCs was independent from the Wnt/β-catenin signaling [50]. Long-term treatment of ESCs with the PI3K inhibitor LY294002 was shown to inhibit the proliferation and to induce cell cycle arrest at the G1 phase [77]. The PI3K/AKT is known to control cell-cycle regulation, where AKT promotes the G1/S-phase transition by facilitating the formation of cyclin/CDK complexes [3]. ESCs lack cell-cycle inhibitory mechanisms, which are typical of differentiated cells. They acquire these mechanisms only upon withdrawal of LIF and consequent differentiation of the cells [81]. It is therefore possible that the AKT-mediated maintenance of self-renewal in ESCs is due to its ability to block the cell-cycle inhibitory mechanisms and consequently block differentiation [50]. Recently, it was shown that AKT1 interacts with and phosphorylates SOX2 at a highly conserved threonine residue in self-renewing mouse ESCs. This modification leads to an enhanced transcriptional activity and to an increase in SOX2 protein which accumulates in the nucleus [82].

### 2.3. LIF/STAT3 and Reprogramming

Genome-wide epigenetic reprogramming occurs during normal mammalian development and consists in the erasure and remodeling of epigenetic marks, such as DNA methylation. This process is typically linked to the reversal of differentiation and can be exploited to re-establish the pluripotent state typical of ESCs. In the past years several reprogramming systems have been developed to restore pluripotency in somatic cells. The two most widely used methodologies are the somatic nuclear transfer (SCNT) and the generation of induced pluripotent stem cells (iPSCs). The first technology consists in the transfer of a somatic nucleus into an oocyte. Oocytes or zygotes are able to erase epigenetic information from the somatic nucleus [83–85]. Reconstituted embryos are able to proceed through the cleavage stages into the blastocyst stage. The success rate of SCNT is nevertheless extremely low. A more elegant way of reprogramming was recently introduced by Takahashi and Yamanaka [34,86,87]. This technology consists in the transfection of differentiated cells with the master transcriptional regulators *Oct3/4* (*Pou5f1*) and *Sox2*, in combination with *c-Myc* and *Klf4*. This work was followed by a number of further publications from different laboratories that contributed to various improvements of the original protocol. Meanwhile several different combinations of transcription factors have been shown to be sufficient to induce reprogramming. For example, the combinations of *Nr5a2*, *Sox2*, *Klf4*, and *c-Myc* [88] or *Oct3/4*, *Nanog*, *Klf4*, and *c-Myc* or *Oct4*, *Sox2*, *Nanog*, and *Lin28* [89] were described. Nevertheless, the *in vitro* reprogramming process is still very inefficient and requires considerably more time than the reprogramming processes that occur *in vivo*. Further analysis of the mechanisms underlying reprogramming *in vivo* and *in vitro* are needed in order to improve this technology.

After transduction of the reprogramming factors and throughout the reprogramming process, the cells need to be maintained in the presence of LIF. Until recently it was unclear if LIF and its downstream target STAT3 maximize the self-renewal of the newly generated iPSCs or do have an active role in the reprogramming process. In a recent work, Yang and colleagues demonstrated that STAT3 cooperates with the reprogramming factors and directly contributes to the induction and reactivation of the endogenous pluripotency network to generate iPSCs [90]. The authors of this study used an engineered chimeric GCSFR/gp130 receptor that combines the binding domain of the granulocyte colony-stimulating factor (GCSF) receptor with the transmembrane and a mutated cytoplasmic domain of the LIFR. This chimeric receptor lacks the binding site for the negative feedback regulator SOCS3 and can signal only via JAK/STAT3 activation and not through the MAPK/ERK- and PI3K-pathways. GCSF-induced STAT3 activation was shown to promote not only epiblast stem cell (EpiSC) to ESC conversion but also to increase the efficiency of generating Oct4-GFP+ cells from the partially reprogrammed iPSC line in cooperation with 2i.

Interestingly, phosphorylation of STAT3 did not result in the immediate induction of the pluripotency factors *Nanog* or *Klf4* suggesting that the contribution of STAT3 in creating a poised state for reprogramming is obtained through the activation of additional target genes, which have not been identified yet.

**Figure 2:**
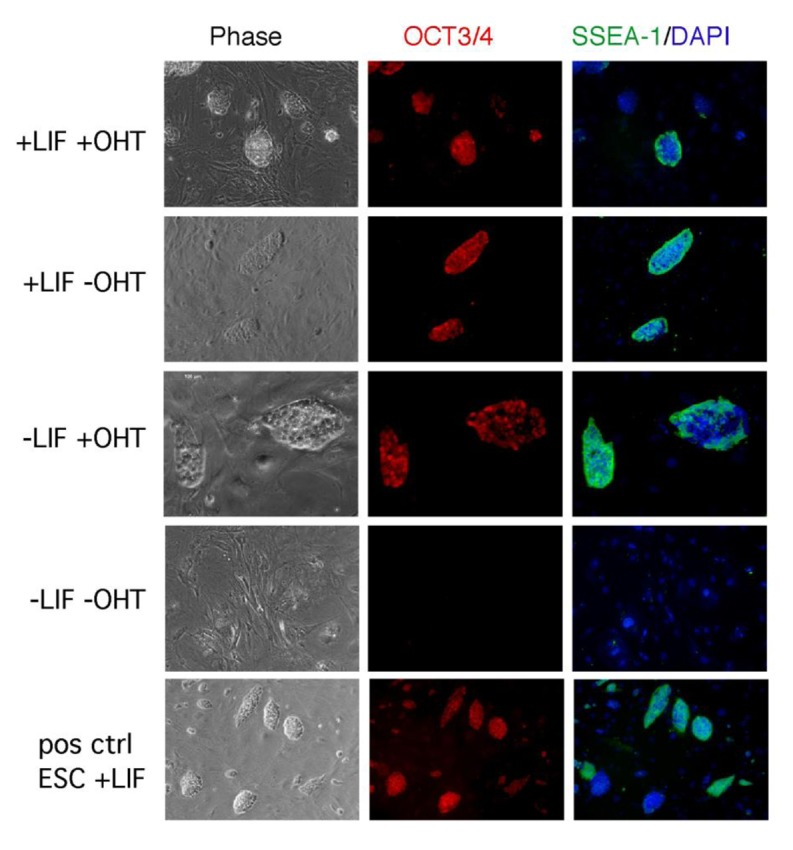
IHC staining of induced pluripotent stem cell (iPSC) clones expressing STAT3MER after 9 days in different culture conditions. iPSCs cultivated on *Lif*−/− feeders either in the presence of LIF, or LIF and OHT, or OHT alone were able to maintain their pluripotent state. Cells cultivated without LIF and OHT differentiated. Positive control: E14 ESCs cultivated in presence of LIF. Scale bar: 100 μm for 20× and 200 μm for 10× pictures.

The role of LIF/STAT3 is still elusive also in the maintenance of iPSCs. In ESCs, the overexpression of STAT3 is sufficient to maintain ESCs pluripotent in the absence of LIF [18]. We generated iPSCs from fibroblasts overexpressing a tamoxifen inducible form of STAT3 (STAT3MER) and determined the iPSC-stability in culture by cultivating the cells under four different culture conditions (+LIF+OHT/+LIF−OHT/−LIF+OHT/−LIF−OHT). As expected, the cells cultivated in the presence of LIF were able to self-renew, whereas iPSCs maintained in the absence of LIF differentiated. Interestingly cells cultivated in medium supplemented with OHT but not LIF, were able to maintain their pluripotent state, as shown by SSEA-1 and OCT3/4 immunohistochemical analysis (Figure 2) and alkaline phosphatase activity. These findings clearly highlight the similarity between ESCs and iPSCs in respect to their LIF/STAT3 dependence.

## 3. Experimental Section

### 3.1. Generation and Cultivation of STAT3-MER iPSCs

iPSCs cells were obtained by retroviral infection of STAT3MER mouse embryonic fibroblasts (MEFs) obtained from FVB/N STAT3MER (Tg743) [58] by using the pMXs-based retroviral reprogramming system [86,87]. Wild type and STAT3MER- expressing iPSC clones were seeded on LIF−/−feeders and cultivated in medium with or without LIF and in the presence or absence of 4-hydroxy tamoxifen (Sigma). After 9 days in culture, cells were fixed and stained for pluripotency markers SSEA-1 and OCT3/4.

### 3.2. Immunohistochemical Analysis of STAT3-MER iPSCs

ESCs were fixed in 4% paraformaldehyde and incubated overnight at 4 °C with primary antibodies against OCT3/4 (rabbit anti OCT3/4, Santa Cruz Biotechnology) and SSEA-1 (Mouse mAb, Chemicon International). Secondary fluorescence labeled antibodies were used for detection (anti rabbit Alexa Fluor 594 and anti mouse Alexa Fluor 488, Molecular Probes). Nuclei of the cells were counterstained with DAPI (Roche).

## 4. Conclusions

In this review article, we summarized the actual knowledge on the importance of LIF in the maintenance of pluripotency and self-renewal in embryonic and induced pluripotent stem cells. The work performed over the past two decades by different research groups all over the world has identified the LIF-pathway as a crucial element for the regulation of self-renewal and for the maintenance of pluripotency in ESCs and iPSCs. Nevertheless, even though the LIF-mediated pathways in ESCs were extensively studied the precise mechanisms how they are regulated still remain unclear. Especially the role of STAT3 in reprogramming requires further analysis. The fact that *Stat3* activation during reprogramming does not result in the immediate induction of its known target genes is of interest, suggesting that the contribution of STAT3 in this process is obtained through the regulation of additional target genes. The identification of these genes and the further characterization of the mechanisms involved in maintenance of stem cells will be an important goal for the future. These studies are of major interest because ESCs and iPSCs have great potential for therapeutic applications, especially for tissue engineering, regenerative medicine and cell therapy.
